# Natural quasicrystal with decagonal symmetry

**DOI:** 10.1038/srep09111

**Published:** 2015-03-13

**Authors:** Luca Bindi, Nan Yao, Chaney Lin, Lincoln S. Hollister, Christopher L. Andronicos, Vadim V. Distler, Michael P. Eddy, Alexander Kostin, Valery Kryachko, Glenn J. MacPherson, William M. Steinhardt, Marina Yudovskaya, Paul J. Steinhardt

**Affiliations:** 1 Dipartimento di Scienze della Terra, Università di Firenze, Via La Pira 4, I-50121 Florence, Italy; 2 Princeton Institute for the Science and Technology of Materials, Bowen Hall, Princeton University, Princeton, NJ 08544, USA; 3 Department of Physics, Princeton University, Jadwin Hall, Princeton, NJ 08544, USA; 4 Department of Geosciences, Princeton University, Guyot Hall, Princeton, NJ 08544, USA; 5 Division of Earth and Atmospheric Sciences, Purdue University, West Lafayette, IN 47907, USA; 6 Institute of Geology of Ore Deposits, Petrography, Mineralogy, and Geochemistry (IGEM), Russian Academy of Sciences, Staromonetny per. 35, Moscow, 119017 Russia; 7 Department of Earth, Atmospheric, and Planetary Sciences, Massachusetts Institute of Technology, Cambridge, MA 02139, USA; 8 Geoscience Technology, BHP Billiton, Houston, TX 77056, USA; 9 Department of Mineral Sciences, National Museum of Natural History, Smithsonian Institution, Washington DC. 20013, USA; 10 Department of Earth and Planetary Sciences, Harvard University, 20 Oxford Street, Cambridge, MA 02138, USA; 11 Princeton Center for Theoretical Science, Princeton University, Princeton, NJ 08544 USA

## Abstract

We report the first occurrence of a natural quasicrystal with decagonal symmetry. The quasicrystal, with composition Al_71_Ni_24_Fe_5_, was discovered in the Khatyrka meteorite, a recently described CV3 carbonaceous chondrite. Icosahedrite, Al_63_Cu_24_Fe_13_, the first natural quasicrystal to be identified, was found in the same meteorite. The new quasicrystal was found associated with steinhardtite (Al_38_Ni_32_Fe_30_), Fe-poor steinhardtite (Al_50_Ni_40_Fe_10_), Al-bearing trevorite (NiFe_2_O_4_) and Al-bearing taenite (FeNi). Laboratory studies of decagonal Al_71_Ni_24_Fe_5_ have shown that it is stable over a narrow range of temperatures, 1120 K to 1200 K at standard pressure, providing support for our earlier conclusion that the Khatyrka meteorite reached heterogeneous high temperatures [1100 < *T*(K) ≤ 1500] and then rapidly cooled after being heated during an impact-induced shock that occurred in outer space 4.5 Gya. The occurrences of metallic Al alloyed with Cu, Ni, and Fe raises new questions regarding conditions that can be achieved in the early solar nebula.

The first and, until now, the only known natural quasicrystal, Al_63_Cu_24_Fe_13_ (icosahedrite), exhibits icosahedral symmetry and was reported in 2009 as grains within a rock sample found in the Museo di Storia Naturale of the Università degli Studi di Firenze (catalog number 46407/G), labeled as khatyrkite and identified as coming from the Khatyrka ultramafic zone in the Koryak Mountains in far eastern Russia^1^,^2^,^3^. This sample contains a metallic core consisting primarily of icosahedrite, khatyrkite (CuAl_2_), cupalite (CuAl), and β-phase (AlCuFe) intergrown with diopside, forsterite, and minute amounts of stishovite. The core is encased in a white rind that includes spinel, pyroxene ranging in composition from diopside to hedenbergite, nepheline, and sodalite. The composition of icosahedrite was found to closely match that of the first stable quasicrystal synthesized in the laboratory^4^.

The rock sample was subsequently shown to be a fragment of the Khatyrka meteorite, an oxidized subgroup (Allende-like) CV3 carbonaceous chondrite (CV3 CC), which formed at least 4.5 billion years ago^5^. Additional samples of the Khatyrka meteorite were recovered in an expedition to the Koryak Mountains in Chukotka in 2011^6^,^7^. A study of those samples provided clear evidence that the meteorite was subjected to a high-pressure shock and that the shock produced a heterogeneous distribution of high pressure and temperature followed by rapid cooling^8^. Studies of the recovered samples continue to reveal information about the mineral phases and the assemblages that resulted from the impact.

This paper reports the discovery of a second natural quasicrystal. The quasicrystal was identified in a powder sample from Grain 126 of the same Khatyrka meteorite, as described by Hollister et al.^8^ The quasicrystal has composition Al_71_Ni_24_Fe_5_ and is the first known natural quasicrystal with decagonal symmetry, a periodic stacking of layers containing quasiperiodic atomic arrangements with ten-fold symmetry. The first decagonal quasicrystal was synthesized in the laboratory in 1985 in rapidly quenched Al-Mn samples^9^,^10^,^11^. A decagonal phase in the Al-Ni-Fe system was first synthesized, again by rapid solidification, in 1989 by Tsai et al.^12^ Lemmerz et al.^13^ later identified the stability range to be centered around composition Al_71_Ni_24_Fe_5_ – consistent with the composition of the natural quasicrystal – over a narrow range of temperatures between 1120 K and 1200 K. This supports our earlier conclusion^8^ that the Khatyrka meteorite reached heterogeneous high temperatures and then rapidly quenched after undergoing an impact-induced shock that occurred in outer space at the beginning of the solar system.

Quasicrystals^14^,^15^ are novel phases of matter easily recognized by their quasiperiodic atomic arrangements (i.e., described by a sum of two or more periodic functions whose periods have an irrational ratio) and by their rotational symmetries forbidden to periodic crystals, including five-fold and ten-fold symmetry axes. More than one hundred different compositions of quasicrystals have since been synthesized in the laboratory^16^, many of which including metallic Al. Until now, though, Khatyrka is the only meteorite found to contain phases with metallic Al as an essential component. The existence of icosahedrite and the decagonal quasicrystal with metallic Al imply that remarkably low oxygen fugacities were achieved during the formation of the meteorite in the early solar system.

## Results

### Description of the sample

**Figure 1:**
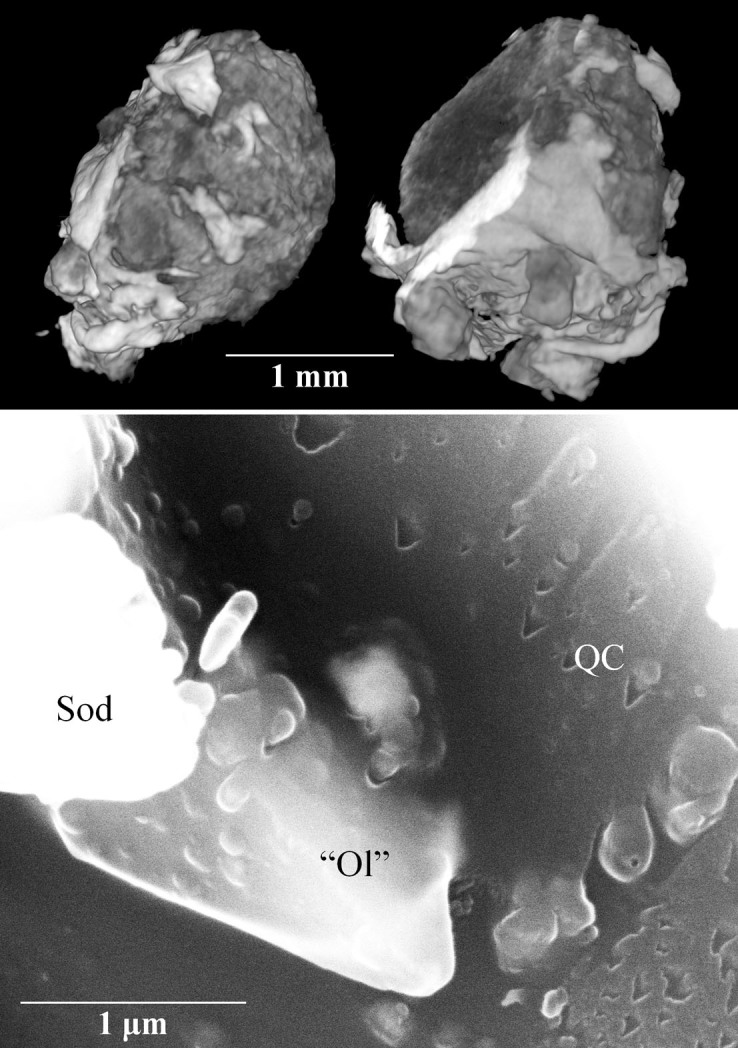
The top panel shows micro CT-SCAN 3D-images (at different rotations) of the whole Grain 126. The brighter and the darker regions are Cu-Al metals and meteoritic silicates, respectively. The bottom panel shows a SEM-BSE image of Al_71_Ni_24_Fe_5_ quasicrystal (QC) in apparent growth contact with “olivine” (“Ol”). See text for discussion of the “olivine” composition. The surface of the quasicrystal appears to exhibit growth steps. The image also contains sodalite (Sod).

Grain 126 is dark grey in incident light with diverse silicate and metallic fragments visible. No fusion crust is preserved on the sample. The fragment has meteoritic (CV3-like) oxygen isotopic compositions^8^. X-ray computed tomography studies showed the presence of a large khatyrkite grain (bright areas in the upper panel of Fig. 1) clearly attached to the meteorite fragment (dark areas in the upper panel of Fig. 1), as typically observed for other fragments of the Khatyrka meteorite^5^,^7^,^8^. Detailed examination by scanning electron microscopy (SEM) and transmission electron microscopy (TEM) of tiny particles from Grain 126 revealed the presence of Al-bearing trevorite, coesite, stishovite, magnetite, diopside, forsterite, clinoenstatite, sodalite, nepheline, pentlandite, Cu-bearing troilite, icosahedrite, khatyrkite, cupalite, taenite, Al-bearing taenite, and the recently approved mineral steinhardtite^17^. The recovery of several intriguing Al-Ni-Fe metallic phases, including the new polymorph of Al named steinhardtite^17^, motivated a careful search for other metallic fragments, which led to the discovery of more steinhardtite grains and the Al-rich fragments with compositions close to that of the known Al-Ni-Fe decagonal quasicrystal.

### Analysis of selected Al-Ni-Fe fragments

**Figure 2:**
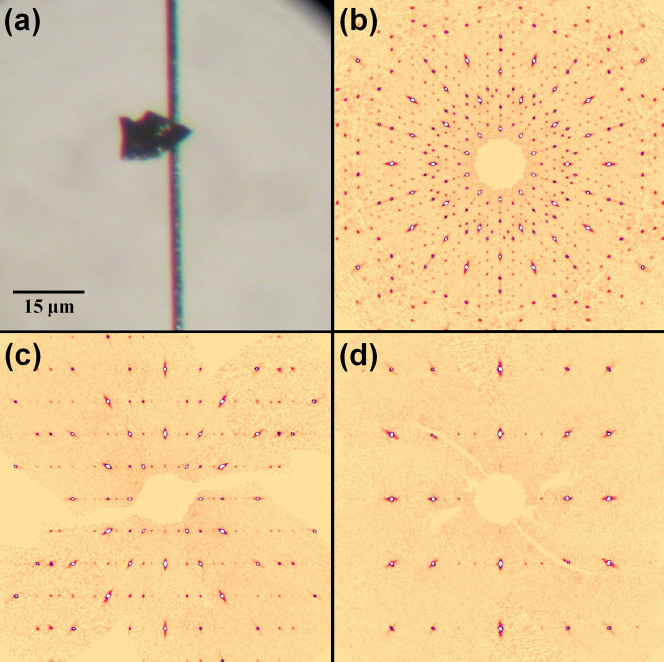
Reconstructed precession images along the ten-fold symmetry axis (b) and perpendicular to the ten-fold direction (c, d) obtained using the collected single-crystal X-ray data set (Mo*K*α radiation) from the fragment of Grain 126 shown in (a).

**Figure 3:**
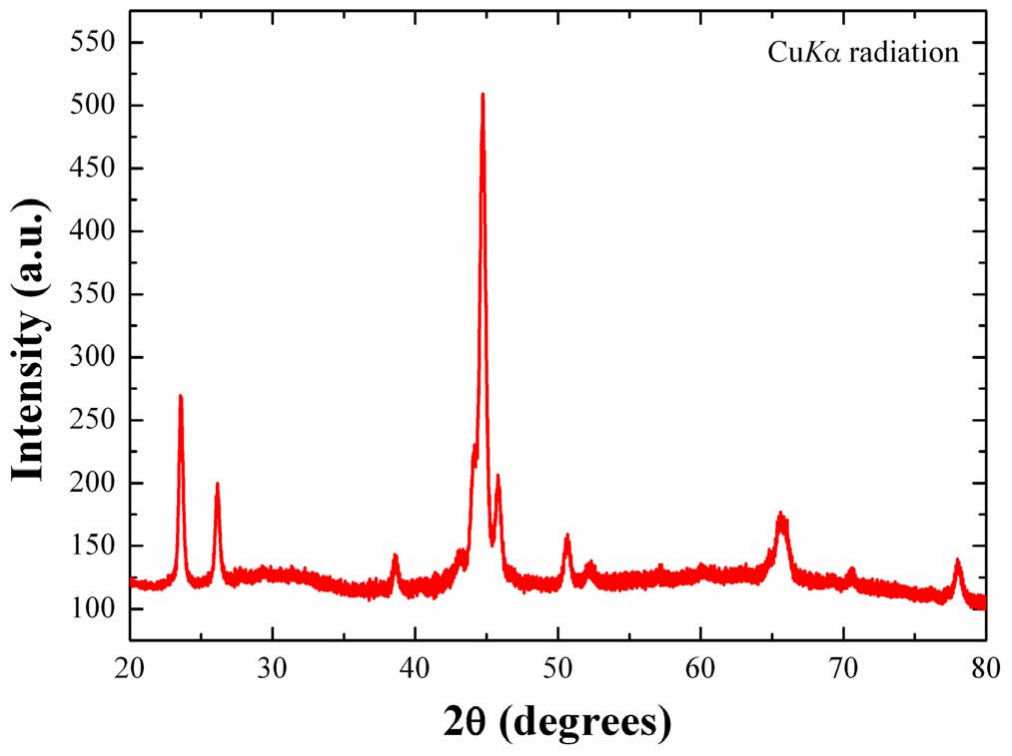
X-ray powder diffraction pattern for natural Al_71_Ni_24_Fe_5_ (Cu*K*α radiation).

The most promising fragments were handpicked from a TEM grid and studied by means of electron microprobe and X-ray diffraction techniques. The chemical data (Table 1) confirmed the presence of some fragments with the same Al_38_Ni_32_Fe_30_ stoichiometry reported for steinhardtite^17^, and indicated the presence of fragments with compositions Al_50_Ni_40_Fe_10_ and Al_71_Ni_24_Fe_5_. Selected samples of both these compositions were then studied by single-crystal X-ray diffraction. The two investigated Al_50_Ni_40_Fe_10_ fragments exhibit the steinhardtite structure (cubic, space group 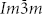) with parameters *a* = 3.065(3) Å and *a* = 3.059(2) Å, and thus can be considered as Fe-poor steinhardtite. The lattice values for the two fragments are slightly larger than that reported for steinhardtite (i.e., *a* = 3.0214(8) Å^17^;), in agreement with the higher Al content^18^. On the other hand, the diffraction analysis of one of the Al_71_Ni_24_Fe_5_ fragments revealed the unmistakable signature of a decagonal quasicrystal: a pattern of sharp peaks arranged in straight lines with ten-fold symmetry together with periodic patterns taken perpendicular to the ten-fold direction (as illustrated in Fig. 2). A second Al_71_Ni_24_Fe_5_ fragment consists of many tiny grains and thus a powder diffraction pattern was collected (Fig. 3). The pattern matches precisely that reported for the synthetic decagonal Al_71_Ni_24_Fe_5_ quasicrystal^12^.

**Figure 4:**
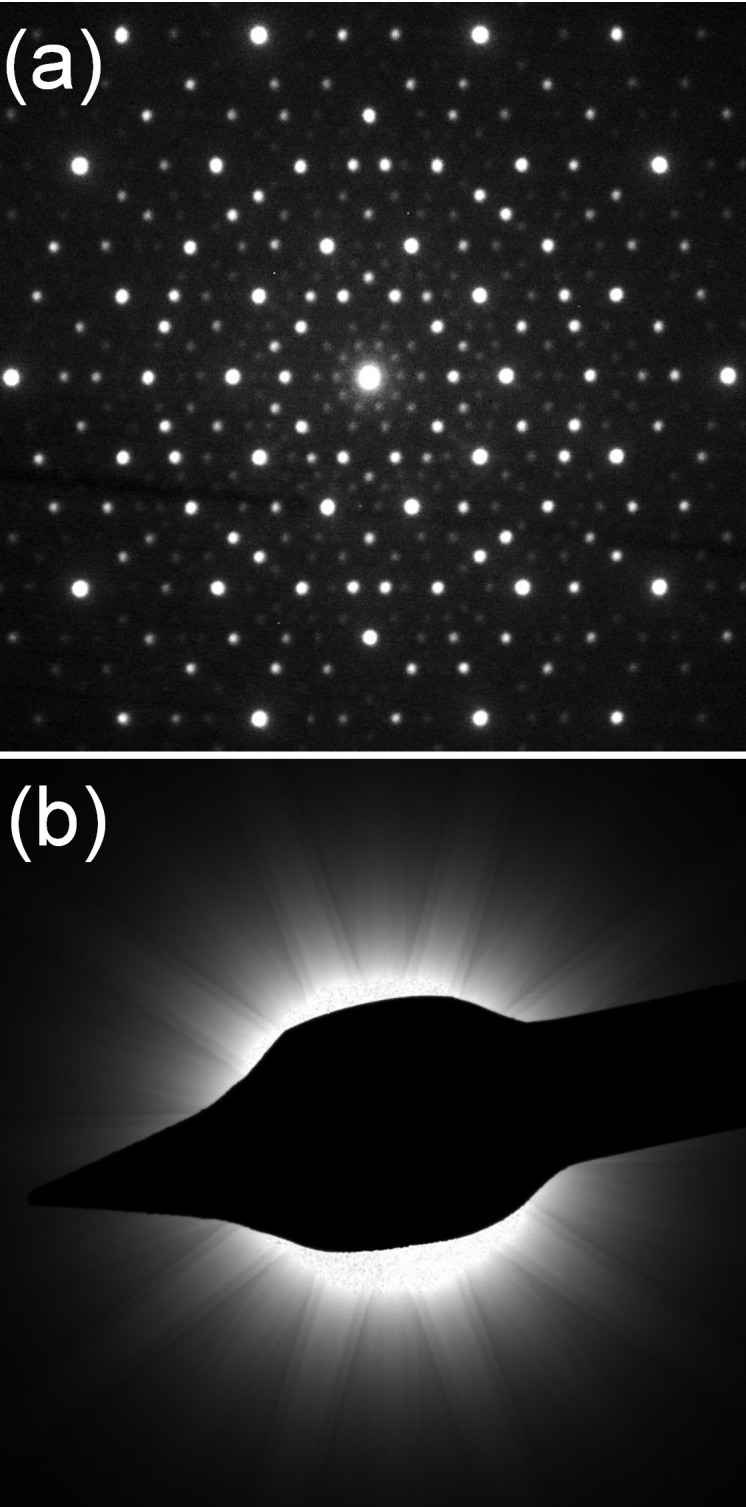
Selected area (a) and convergent beam (b) electron diffraction patterns collected with a TEM along the ten-fold axis. These patterns, consisting of sharp peaks (or Kikuchi lines) arranged with ten-fold symmetry, are the characteristic signature of a decagonal quasicrystal.

**Figure 5:**
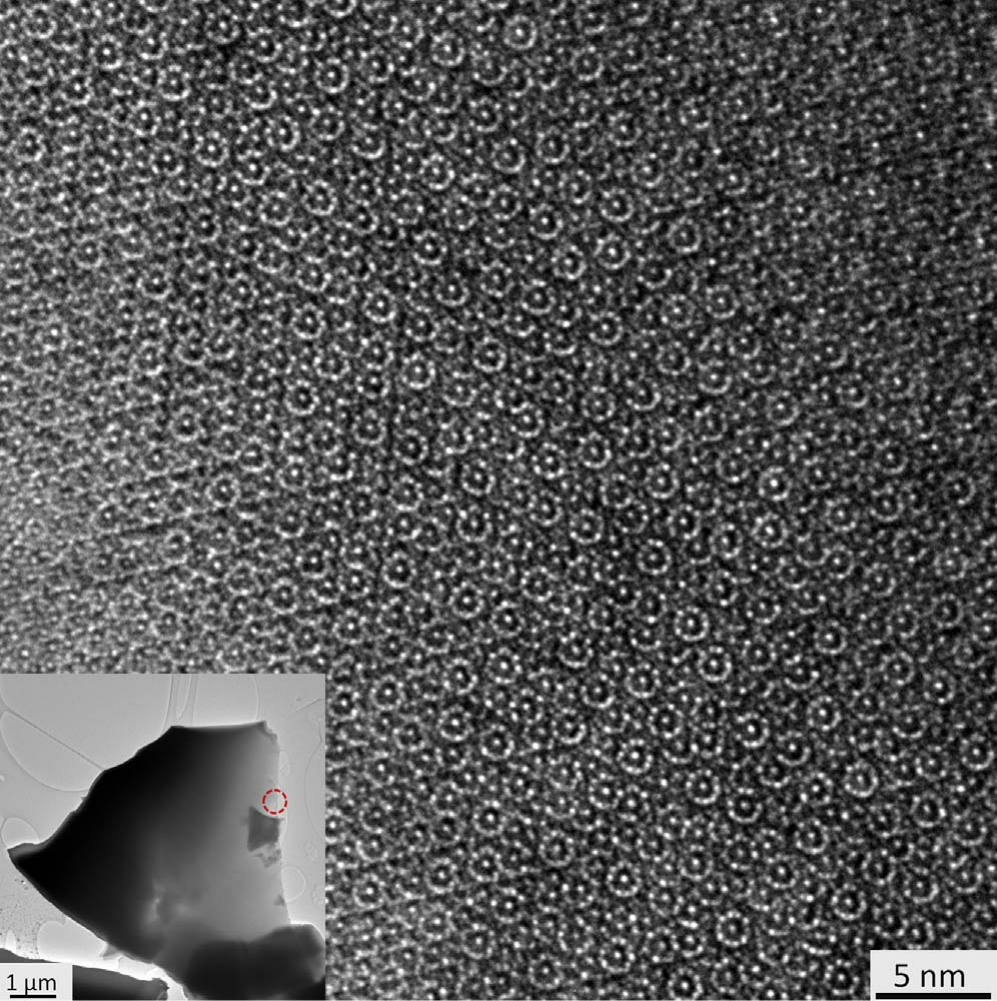
High-resolution transmission electron microscopy (HRTEM) image showing that the real space structure consists of a homogeneous, quasiperiodic and ten-fold symmetric pattern. The diffraction patterns given in Fig. 4 and the HRTEM image were obtained from the thin region of the granule in the inset indicated by the red (dashed) circle, an area 0.2 μm across.

One of the Al_71_Ni_24_Fe_5_ fragments tested by X-ray was crushed and placed on a TEM grid (300 mesh, 3 mm in diameter) and the largest one (80 × 25 × 10 μm in size) was used to study the metal-silicate contacts by scanning electron microscopy. The TEM study revealed that at the sub-micron length scale, the grains are completely homogeneous. Selected area and convergent electron beam diffraction patterns along the ten-fold axis are shown in Fig. 4a and 4b. These patterns, consisting of sharp peaks (or Kikuchi lines) arranged in an incommensurate lattice with ten-fold symmetry, are the characteristic signature of a decagonal quasicrystal. The high-resolution transmission electron microscopy image in Fig. 5 shows that the real space structure consists of a homogeneous, quasiperiodic and ten-fold symmetric pattern. Together, these TEM results provide conclusive evidence of crystallographically forbidden decagonal symmetry in a naturally occurring phase.

The SEM study of the largest Al_71_Ni_24_Fe_5_ fragment shows a clear contact with a (Fe,Mg)_2_SiO_4_ phase (marked “Ol” in the bottom panel of Fig. 1). This is either an intermediate composition olivine similar to the Fo_45–50_ found in Grain 125 or the high-pressure polymorph ahrensite, which was also observed in Grain 125^8^. Also shown is a grain of sodalite (marked Sod).

## Discussion

The discovery of a decagonal quasicrystal, Al_71_Ni_24_Fe_5_, is notable for several reasons. It is only the second example of a natural quasicrystal to be found anywhere, and it is the first example of a natural quasicrystal with decagonal symmetry. The sample occurs in the Khatyrka meteorite, the first meteorite observed to contain a spectrum of metal alloy minerals with metallic aluminum, including the Al-bearing quasicrystals icosahedrite and the decagonal quasicrystal here described. We note here, again, that many synthetic quasicrystals contain metallic Al.

The processes that produced the conditions leading to the formation of phases with metallic Al are still unknown. Hollister et al.^8^ suggested two possibilities: (*i*) Al-bearing taenite was the initial source of aluminum that reacted with Cu and Fe after a high-velocity impact to form the variety of observed Al-bearing minerals; or (*ii*) the aluminum originated in Cu-Al-metals that had a pre-accretion nebular origin. We are currently collecting data for evaluating these and perhaps additional conjectures. Whatever the process, it must be that, once metallic Al is produced in an iron-rich environment, a wide range of Al- Cu- and Fe-bearing phases can form: including the two quasicrystals, steinhardtite (Al_38_Ni_32_Fe_30_), Fe-poor steinhardtite (Al_50_Ni_40_Fe_10_), Al-bearing trevorite (NiFe_2_O_4_) and Al-bearing taenite (FeNi).

Another feature the two natural quasicrystals have in common is the high degree of structural perfection, particularly the absence of significant phason strains. Phasons are elastic modes that relax diffusively and are commonly frozen-in during rapid quenches of laboratory samples. The characteristic signature of phason disorder is a systematic shift in the Bragg peak positions from the ideal by an amount that increases as the peak intensity decreases. The apparent absence of phason strain in the diffraction patterns (Fig. 4a) for the natural decagonal quasicrystal suggests a slow quench. This conclusion, though, is somewhat at odds with the other evidence suggesting that the melt produced by the impact-induced shock reached a temperature near 1500 K and then rapidly quenched. The rapid quench was invoked, in part, to explain why there are not significant oxidation reaction rims at the interface between Al-bearing metallic phases and silicates or oxides. Furthermore, a difference between the two quasicrystals is that icosahedrite at the observed composition is stable at standard conditions, whereas the decagonal phase is only stable in the temperature range 1120 K to 1200 K, at least at standard pressure. The limited temperature range of stability for the decagonal quasicrystal also supports the interpretation of rapid quench. An important consideration, though, may be the pressure because the evidence also suggests that impact produced an unusually high pressure for the CV3 carbonaceous chondrites, as much as 5–10 GPa, in some regions of the sample. At high pressures, perhaps the reducing, stability and quench conditions are relaxed. Our further studies of Khatyrka meteorite samples and our high pressure laboratory studies are aimed at taking proper account of the combination of temperature and pressure conditions to resolve the mystery of how this remarkable assemblage of minerals formed and its implications for processes in the early solar system.

## Methods

### Sample characterization techniques

The sample studied here (Grain 126) was investigated by means of micro-CT (computed tomography), TEM (transmission electron microscopy), SEM-EDS (scanning electron microscopy, energy dispersive spectrometry) and EMP-WDS (electron microprobe, wavelength dispersive spectrometry) techniques.

### X-ray computed tomography

The instrument was a micro-CT SkyScan 1172 equipped with a 11Mpixel detector with a resolution of 0.8 μm and operating at 80 kV (X-ray tungsten radiation) with a spot size of 0.3 μm.

### Transmission electron microscopy

A small amount of powder from the sample was placed on a Cu mesh TEM grid (300 mesh, 3 mm in diameter) that was previously covered by a thin carbon layer (support film). EDS data were obtained using Evex NanoAnalysis System IV attached to the Philips CM200-FEG TEM. A small electron probe of 20–100 nm was used with a count rate of 100–300 cps using an average collection time of 180 s. The quantitative analyses were taken at 200 kV and are based on using pure elements and the NIST 2063a standard sample as a reference under the identical TEM operating conditions. Another JEOL JEM 2010 (operating at 200 kV with an ultra-high resolution (UHR) pole piece, and a point-to-point resolution close to 1.9 Å) was also used.

### Scanning electron microscopy

The same powder studied with the TEM was then analyzed by means of a FEI Quanta 200 FEG Environmental-Scanning Electron Microscope equipped with an Oxford INCA Synergy 450 energy-dispersive X-ray microanalysis system, operated at 15 kV and 5 kV accelerating voltage, 140 pA probe current, 2,000 cps as average count rate on the whole spectrum, and a counting time of 60 s, and with a Zeiss - EVO MA15 Scanning Electron Microscope coupled with an Oxford INCA250 energy-dispersive spectrometer, operating at 20 kV and 5 kV accelerating voltage, 500–150 pA probe current, 2,500 cps as average count rate on the whole spectrum, and a counting time of 500 s. The lower voltages were used in order to minimize secondary radiation from adjacent phases.

### Electron microprobe

All the available Al-Ni-Fe-bearing fragments with suitable size present in the powder on the TEM grid (three Al_38_Ni_32_Fe_30_, three Al_50_Ni_40_Fe_10_ and three Al_71_Ni_24_Fe_5_ grains) were handpicked and studied with a JEOL JXA-8600 electron microprobe operating at an accelerating voltage of 15 kV, beam current of 20 nA, and a beam diameter of 1 μm. Variable counting times were used: 30 s for Al, Ni and Fe, and 60 s for the minor elements Mg, Si, Cr, P, Co, Cu, Cl, Ca, Zn, and S. Replicate analyses of synthetic Al_53_Ni_42_Fe_5_ were used to check accuracy and precision. The crystal fragments were found to be homogeneous within analytical error (see Table 1). The standards used were: metal-Al (Al), synthetic Ni_3_P (Ni, P), synthetic FeS (Fe), metal-Mg (Mg), metal-Si (Si), metal-Cr (Cr), metal-Co (Co), metal-Cu (Cu), synthetic CaCl_2_ (Ca, Cl) and synthetic ZnS (Zn, S). Magnesium, Si, Cr, P, Co, Cu, Cl, Ca, Zn, and S were checked and found to be equal to or below the limit of detection (0.05 wt%).

### X-ray diffraction

When the chemical analyses obtained at the microprobe showed similarities with those of known quasicrystals (i.e., decagonal Al_71_Ni_24_Fe_5_), individual samples of each metallic phase with its surrounding material were extracted to perform X-ray diffraction studies. Such studies were done with both an Oxford Diffraction Xcalibur 3 CCD single-crystal diffractometer, operating with Mo*K*α radiation (λ = 0.71073 Å), and an Oxford Diffraction Excalibur PX Ultra diffractometer equipped with a 165 mm diagonal Onyx CCD detector at 2.5:1 demagnification operating with Cu*K*α radiation (λ = 1.5406 Å).

## Author Contributions

The study was conceived and guided by L.B., L.S.H., G.J.M. and P.J.S., who also led the research team. L.B., N.Y., C.L., L.S.H. and P.J.S. performed the SEM and electron microprobe studies. L.B. performed the micro-CT (computed tomography) and single-crystal and powder X-ray diffraction studies. N.Y. performed the TEM studies. L.B., C.L.A., G.J.M., V.V.D., M.P.E., A.K., V.K., W.M.S., M.Y. and P.J.S. participated in the scientific expedition to Chukotka in 2011 and helped to recover the new samples. L.B. and P.J.S. wrote the paper. All the authors discussed the results and commented on the manuscript.

## Figures and Tables

**Table 1:** Electron microprobe analyses (wt% of elements – standard deviations in parentheses) and atomic ratios, on the basis of 100 atoms, for Al-Ni-Fe-bearing metallic phases in Grain 126

*Steinhardtite fragments*								
	1a	1b	1c	2a	2b	2c	3a	3b
Al	21.94(12)	22.41(15)	22.19(21)	22.11(10)	22.65(13)	22.67(15)	21.90(18)	22.13(24)
Ni	40.68(33)	40.01(28)	40.18(33)	41.02(22)	42.10(26)	41.55(29)	41.31(33)	41.44(38)
Fe	36.22(41)	36.57(39)	36.03(35)	37.29(26)	36.79(31)	36.11(29)	36.50(41)	36.08(45)
Total	98.84	98.99	98.40	100.42	101.54	100.33	99.71	99.65
Al	37.74	38.33	38.21	37.48	37.89	38.28	37.42	37.76
Ni	32.16	31.45	31.81	31.97	32.37	32.26	32.45	32.50
Fe	30.10	30.22	29.98	30.55	29.74	29.46	30.13	29.74

*Fe-poor steinhardtite fragments*						
	4a	4b	5a	5b	6a	6b
Al	31.52(40)	31.70(38)	30.90(31)	31.52(34)	32.12(40)	31.93(42)
Ni	54.98(61)	55.25(55)	56.10(44)	55.01(47)	55.23(53)	55.08(50)
Fe	13.85(12)	13.56(13)	13.41(11)	13.02(13)	13.60(15)	13.82(12)
Total	100.35	100.51	100.41	99.55	100.95	100.83
Al	49.65	49.81	48.92	49.95	50.13	49.95
Ni	39.81	39.90	40.82	40.08	39.62	39.60
Fe	10.54	10.29	10.26	9.97	10.25	10.45

*Decagonal quasicrystal*								
	7a	7b	8a	8b	9a	9b	9c	mean
Al	52.23(60)	51.74(64)	52.01(71)	51.60(66)	52.10(44)	52.64(40)	53.01(46)	52.19
Ni	39.85(51)	38.92(49)	40.45(53)	39.41(55)	40.01(34)	39.23(39)	39.01(37)	39.55
Fe	8.02(10)	8.74(12)	7.55(14)	8.23(15)	8.10(9)	8.16(12)	8.47(11)	8.18
Total	100.10	99.40	100.01	99.24	100.21	100.03	100.49	99.92
Al	70.18	70.06	70.05	70.02	70.03	70.55	70.65	70.22
Ni	24.61	24.22	25.04	24.59	24.71	24.17	23.90	24.46
Fe	5.21	5.72	4.91	5.39	5.26	5.28	5.45	5.32
